# The Psychological and Social Impact of Covid-19: New Perspectives of Well-Being

**DOI:** 10.3389/fpsyg.2020.577684

**Published:** 2020-10-02

**Authors:** Valeria Saladino, Davide Algeri, Vincenzo Auriemma

**Affiliations:** ^1^Department of Human Sciences, Society and Health, University of Cassino and Southern Lazio of Cassino, Cassino, Italy; ^2^Independent Researcher, Milan, Italy; ^3^Department of Political and Social Studies, Sociology, University of Salerno, Fisciano, Italy

**Keywords:** COVID-19, empathy, psychological disease, psychotherapy, social distancing, telepsychology

## Abstract

The recent Covid-19 pandemic has had significant psychological and social effects on the population. Research has highlighted the impact on psychological well-being of the most exposed groups, including children, college students, and health workers, who are more likely to develop post-traumatic stress disorder, anxiety, depression, and other symptoms of distress. The social distance and the security measures have affected the relationship among people and their perception of empathy toward others. From this perspective, telepsychology and technological devices assume important roles to decrease the negative effects of the pandemic. These tools present benefits that could improve psychological treatment of patients online, such as the possibility to meet from home or from the workplace, saving money and time and maintaining the relationship between therapists and patients. The aim of this paper is to show empirical data from recent studies on the effect of the pandemic and reflect on possible interventions based on technological tools.

## Introduction

The Covid-19 pandemic led to a prolonged exposure to stress. As a consequence, researchers showed an increased interest in measuring social and community uneasiness in order to psychologically support the population. This increased attention might help in managing the current situation and other possible epidemics and pandemics. The security measures adopted in managing the pandemic had different consequences on individuals, according to the social role invested. Some segments of the population seem to be more exposed to the risk of anxious, depressive, and post-traumatic symptoms because they are more sensitive to stress.

The following article has two focuses of interest: (1) the evaluation of the psychological and social effects of the pandemic on the population, mostly children, college students, and health professionals; and (2) the identification of new perspectives of intervention based on digital devices and in line with the social security measures and mental health promotion. Telepsychology, for instance, is a valid tool, effective in taking charge of the psychological suffering caused by the pandemic and in preventing the chronicity of the disease. The prolonged stress could involve anxiety, depression, and the inability to manage traumatic and negative emotions. Furthermore, the constant fear of contagion affects daily life and leads to social isolation, modifying human relations.

## COVID-19 and At-Risk Populations: Psychological and Social Impact of the Quarantine

Studies of pandemics faced over time, such as SARS, Ebola, H1N1, Equine Flu, and the current COVID-19, show that the psychological effects of contagion and quarantine is not limited on the fear of contracting the virus (Barbisch et al., 2015). There are some elements related to the pandemic that affect more the population, such as separation from loved ones, loss of freedom, uncertainty about the advancement of the disease, and the feeling of helplessness (Li and Wang, 2020; Cao et al., 2020). These aspects might lead to dramatic consequences (Weir, 2020), such as the rise of suicides (Kawohl and Nordt, 2020). Suicidal behaviors are often related to the feeling of anger associated with the stressful condition widely spread among people who lived/live in the most affected areas (Miles, 2014; Suicide Awareness Voices of Education, 2020; Mamun and Griffiths, 2020). In light of these consequences, a carefully evaluation of the potential benefits of the quarantine is needed, taking into account the high psychological costs (Day et al., 2006; Mazza et al., 2020).

As reported in a recent survey administered during the Covid-19 pandemic, children and young adults are particularly at risk of developing anxious symptoms (Orgilés et al., 2020). The research involved a sample of 1,143 parents of Italian and Spanish children (range 3–18). In general, parents observed emotional and behavioral changes in their children during the quarantine: symptoms related to difficulty concentrating (76.6%), boredom (52%), irritability (39%), restlessness (38.8%), nervousness (38%), sense of loneliness (31.3%), uneasiness (30.4%), and worries (30.1%). From the comparison between the two groups—Spanish and Italian parents—it emerged that the Italian parents reported more symptoms in their children than the Spanish parents. Further data collected on a sample of college students at the time of the spread of the epidemic in China showed how anxiety levels in young adults are mediated by certain protective factors, such as living in urban areas, the economic stability of the family, and cohabitation with parents (Cao et al., 2020). On the contrary, having infected relatives or acquaintances leads to a worsening in anxiety symptoms. Furthermore, the economic problems and the slowdown in academic activities are related with anxious symptoms (Alvarez et al., 2020). In addition, an online survey conducted on the general population in China found that college students are more likely to experiencing stress, anxiety, and depression than others during the pandemic (Li et al., 2020). These results suggest monitoring and promoting mental health of youths in order to reduce the negative impact of the quarantine (CSTS, 2020; Fessell and Goleman, 2020; Li et al., 2020).

Health-care workers (HCWs) are another segment of population particularly affected by stress (Garcia-Castrillo et al., 2020; Lai et al., 2020). HCWs are at risk to develop symptoms common in catastrophic situations, such as post-traumatic stress disorder, burnout syndrome, physical and emotional exhaustion, depersonalization, and dissociation (Grassi and Magnani, 2000; Mache et al., 2012; Øyane et al., 2013). However, an epidemic presents different peculiarities compared to a catastrophic event, for instance, the stigmatizing attitudes in particular toward health professionals, who are in daily contact with the risk of infection (Brooks et al., 2020). During SARS, up to 50% of health-care professionals suffered from acute psychological stress, exhaustion, and post-traumatic stress, caused by the fear of contagion of their family members and the prolonged social isolation (Tam et al., 2004; Maunder et al., 2006).

As a consequence of the pandemic, the health professionals who were overworked suffered high level of psychophysical stress (Mohindra et al., 2020). Health professionals also lived/live in daily life a traumatic condition called secondary traumatic stress disorder (Zaffina et al., 2014), which describes the feeling of discomfort experienced in the helping relationship when treatments are not available for all patients and the professional must select who can access them and who cannot (Roden-Foreman et al., 2017; Rana et al., 2020). Data from a survey on 1,257 HCWs who assisted patients in Covid-19 wards and in second- and third-line wards showed high percentages of depression (50%), anxiety (44.6%), insomnia (34%), and distress (71.5%) (Lai et al., 2020). Also, the constant fear of contagion leads to obsessive thoughts (Brooks et al., 2020), increasing the progressive closure of the person and reducing social relationships. In line with these results, Rossi et al. (2020) evaluated mental health outcomes among HCWs in Italy during the pandemic, confirming a high score of mental health issues, particularly among young women and front-line workers. Furthermore, Spoorthy et al. (2020) conducted a review on the gendered impact of Covid-19 and found that 68.7–85.5% of medical staff is composed of women, and the mean age ranged between 26 and 40 years. Also, women are more likely to be affect by anxiety, depression, and distress (Lai et al., 2020; Zanardo et al., 2020). Liang et al. (2020) also found a relation between age and depressive symptoms associated with the pandemic. Indeed, the medical staff at younger ages (<30 years) reports higher self-rated depression scores and more concern about infecting their families than those of older age. Staff > 50 years of age reported increased stress due to patient’s death, the prolonged work hours, and the lack of personal protective equipment. Cai et al. (2020) also found that nurses felt more nervous compared to doctors.

As emerged by the recent literature, the promotion of psychological interventions on the specific population who is more likely to develop pathologies and suffering is needed. The Lancet Global Mental Health Commission’s observation (Patel, 2018) reported that the use of digital technologies can provide mental health interventions in order to reduce anxiety and stress levels and increase self-efficacy (Kang et al., 2020; Xiao et al., 2020).

## Telepsychology: Training and Promotion of Psychological Well-Being

In order to reduce anxiety and depression symptoms widespread among the population, the World Health Organization (2019) and the Centers for Disease Control and Prevention (2020) proposed specific guidelines on the correct use of health protection with the aim to minimize the distress associated with health-care professions.

At the same time, as a consequence of the emerging issues, psychotherapists provided psychological support online, addressing the technological challenge (Greenberg et al., 2020); Liu et al., 2020). In line with the technological progress, professional organizations promoted specific guidelines and policies related to customer protection, privacy, screening, evaluation, and development of self-help products (Duan and Zhu, 2020; Zhou et al., 2020). Technological development in mental health foreshadows future trends that include “smart” mobile devices, cloud computing, virtual worlds, virtual reality, and electronic games in addition to the traditional psychotherapy tools. In this perspective, it is important to help future generations of psychologists and patients to collaborate in the potential growth areas, through education and training on the benefits and effectiveness of telepsychology (Maheu et al., 2012).

Indeed, more awareness of the potentials of the online services is needed, exploring the main differences between the devices (chat, video-audio consultation, etc.) in order to use them in relation to the specific purposes identified by the professional. For example, the Italian Service of Online Psychology conducted a study based on a service of helpdesk on Facebook. This service guided people in asking for psychological help, working on their personal motivation. At the same time, another helpdesk on Skype provided some psychological sessions *via* webcam (Gabri et al., 2015). In this line, telecounseling is a diffuse online method used by counselors and psychologists during the recent pandemic (De Luca and Calabrò, 2020).

One of the future goals of public and private psychological organizations should be the promotion of specific training for psychologists and psychotherapists, with the following aims: (1) developing the basic skills in managing the effects of a pandemic and of emergency situations; and (2) sensitizing patients to online therapeutic relationship, providing the main rules and benefits of the process (Stoll et al., 2020; Joint Task Force for the Development of Telepsychology Guidelines for Psychologists, 2013). On this line, a significant example is the Virginia Commonwealth University (VCU) which proposed PhDs in telepsychology, with the aim of training future psychologists in managing the psychological effects of the pandemic through an online psychology service (Baylor et al., 2019). The service provided by the VCU had been effective in reducing anxiety, depression (Sadock et al., 2017), and hospital recoveries (Lanoye et al., 2017). As shown, telepsychology assumes a key role in the improvement of health care. Online psychological services avoid geographical barriers and are suitable to become a useful integrated tool in addition to traditional psychotherapy (APS, 2020; Perrin et al., 2020).

## Advantages of Psychological Support and Online Psychotherapy

Online psychological services provide several advantages, especially in the current situation of pandemic. First of all, online services help people in a short period of time, reducing the risk of contagion and the strong feeling of anxiety in both psychotherapists and patients, who feel uncomfortable in doing traditional psychotherapy due to the pandemic (Békés and Aafjes-van Doorn, 2020). Furthermore, Pietrabissa et al. (2015) identified some of the main advantages of telepsychology, such as the decrease in waiting for the consultation, because it takes place from home or from the workplace, saving time and expense, less travel and rental costs for the office, for those who provide the service and for those who use it. As reported by the authors, online psychological services facilitate access to people who struggle to find support close to their social environment, avoiding difficulties related to mobility. Also, online services help people who have less confidence in psychotherapy. Indeed, mostly online psychotherapy takes place in one’s comfort zone, facilitating the expression of problems and feelings.

According to the situations, online services could provide a different medium. For instance, the chat is a useful tool to establish a first assessment of a person who feels uncomfortable in using video. Indeed, the online psychotherapy is perceived as more “acceptable.” Suler (2004) defined the term *online disinhibition effect* demonstrating how the web, unlike the real life, leads to the failure of the hierarchical relationship based on dominant-dominated among individuals; this aspect, according to the author, allows a greater sense of freedom in expressing oneself and less concern related to judgment (*ibid*.). Other researchers (Mantovani, 1995; Tosoni, 2004) have integrated to the construct of *online disinhibition effect* the concept of social space, emphasizing the role of the “situation,” of the “social norms” (Brivio et al., 2010, p. 811), of the tools (“artifacts”), and of the cyberplace, which allow different levels of interaction. Each person has a different experience of the network and several levels of disinhibition. For instance, a mild disinhibition could be a person who chooses to ask for help talking with a psychologist about their problems; while a high disinhibition could be represented by flaming, an expression of online bullying or cyberstalking.

Online psychological services should be integrated with the various territorial services in order to provide the patients local references in relation to the specific health and economic needs. Finally, the possibility for the therapist and for the patient to record the sessions *via* chat and in audio/video mode—with the informed consent of the participants (Wells et al., 2015)—provides another useful tool to compare the sessions and to underline the positive outcomes and the effectiveness of the therapeutic process. According to this perspective, online psychological support and psychotherapy become a resource for psychotherapists and patients in a co-build relationship (Algeri et al., 2019).

## Psychological and Social Suffering and the Empathic Process

In analyzing the psychological impact of the quarantine, the importance for individuals to feel integral part of the society emerged, an aspect often undervalued in psychological well-being. Experts of public health believe that social distancing is the better solution to prevent the spread of the virus. However, although it is not possible to predict the duration of the pandemic, we know very well the serious impact of these measures on the society, on relationships and interactions, in particular on the empathic process. In the early 90s, empathy was described as a form of identification in the psychological and physiological states of others. This definition led to a debate between the disciplines of philosophy of psychology and philosophy of the mind (Franks, 2010). Willard Van Orman Quine (1908–2000) renewed attention to the debate on empathy with a thesis on the development of language and mind in the analytical philosophy. According to Quine, the attribution of the so-called intentional states, through which the psychology commonly explains human behavior, is based on empathy (Treccani, 2020) and leads people to attribute beliefs, desires, and perceptions (Quine, 1990, 1992, Pursuit of Truth: Revised Edition, 1992). Analyzing this aspect within the recent situation of the pandemic, an increment of antithetical positions and attitudes could be noticed. On the one hand, people identify themselves with those who suffer (neighbors, friends, relatives who are living stressful events), promoting activities such as the so-called “suspended expenses.” For instance, solidarity and humanitarian activities, food, and medicine delivery for people who are unable to go to the supermarket. On the other hand, there is a part of the population who experiences a feeling of “forced empathy.” This aspect could be also emphasized by the use of technological devices that might lead to a depersonalization of relationships, forcing the sense of closeness, at least virtually. The hyperconnection of feelings becomes a way to reduce the self-isolation and its consequences, representing the contrary of the idea of Durkheim (1858–1917), who considered society as a specific entity, built on social facts (Durkheim, 1922). The sensation “to be forced to feel” could lead people to distance themselves from others after the emergency situation, incrementing social phobias.

Also, human communication is changing. The formal question “how are you?” at the beginning of a conversation is no longer just a formality, as before the pandemic. For example, the relationship between employee and the manager is different, leading to more responsibilities in listening and understanding feelings expressed during the video call, generating a forced reciprocity. Hence, the aforementioned “forced empathy” may be common in this period because the social distance and the emergency situation make people want to be heard and appreciated, and the simple question “how are you?” becomes an anchor to express fears and emotions (Pasetti, 2020).

## Discussion

The Covid-19 pandemic has affected the way people live interpersonal relationships. The lockdown was characterized of a different organization of daily life, with an incrementation of time at home and a reduction of distance through digital devices. This period was also seen as an evolution in the concept of empathy, producing new perspectives in the study of the phenomenon according to a sociological and neurological points of view. Indeed, empathy—defined as the ability to understand and share the feelings of another—involves several elements, such as: (a) social context and historical period of the individual, (b) neurological mechanisms, and (c) psychological and behavioral responses to feelings of others. The neuro-sociological perspective analyzes the mechanisms involved in the empathic process, focusing on human communication and interpersonal relationships (Singer and Lamm, 2009; Decety and Ickes, 2009). Specifically, in this historical period characterized by an increment in the man–machine relationship, neurosociology could become one of the principal sciences for the study of human relations and technology. “We live increasingly in a human–machine world. Anyone who doesn’t understand this, and who is not struggling to adapt to the new environment—whether they like that environment or not—is already being left behind. Adapting to the new, fast-changing, technologically enhanced context is one of the major challenges of our times. And that certainly goes for education” (Prensky, 2012, p. 64).

According to the abovementioned considerations, our suggestion consists in:

*Primary prevention.* Studying the impact of the pandemic toward an at-risk population to reduce symptoms related to stress and providing specific online psychological counseling based on the target (students, medical staff, parents, and teachers).

*Secondary prevention.* Overcoming the limitations of the human interaction based on digital devices: (1) developing new spaces of inter- and intrasocial communication and new tools of support and psychological treatment, reproducing the multisensory experienced during the face-to-face interaction (Virtual Reality, holograms, serious game etc.); (2) training the next generation of psychotherapists in managing online devices and in implementing their adaptive and personal skills; and (3) sensitizing the general population on telepsychology and its advantages.

*Research according to the neurosociological perspective*. Studying human interaction mediated by new technologies and the role of empathy, associating neuroscience, sociology, and psychology.

## Author Contributions

VS, DA, and VA conceptualized the contribution. VS wrote the paper, reviewed the manuscript, and provided the critical revision processes as PI. All authors approved the submission of the manuscript.

## Conflict of Interest

The authors declare that the research was conducted in the absence of any commercial or financial relationships that could be construed as a potential conflict of interest.
